# The role of anesthesia in TKA length of stay: a data-driven analysis using Boruta algorithm

**DOI:** 10.3389/fmed.2026.1740228

**Published:** 2026-03-16

**Authors:** Lianyi Fan, Jiuhu Liu, Lulin Ma, Jiafeng Ren

**Affiliations:** 1 Department of Anesthesiology, Tangdu Hospital, Fourth Military Medical University, Xi’an, Shaanxi, China; 2 Laboratory of Anesthesia and Critical Care Medicine, Department of Anesthesiology, National-Local Joint Engineering Research Centre of Translational Medicine of Anesthesiology, West China Hospital, Sichuan University, Chengdu, China; 3 Department of Neurology, Tangdu Hospital, Fourth Military Medical University, Xi’an, Shaanxi, China

**Keywords:** Boruta algorithm, general anesthesia, intravertebral anesthesia, length of hospital stay, total knee arthroplasty

## Abstract

**Background:**

Optimizing the length of hospital stay (LOHS) after total knee arthroplasty (TKA) is essential for improving healthcare efficiency. The effect of anesthesia method on LOHS remains controversial and challenging to accurately assess using conventional statistics. This study leverages robust machine learning with dedicated feature selection to clarify its impact within a limited but well-characterized patient cohort.

**Methods:**

We analyzed data from 157 patients undergoing primary unilateral TKA. The Boruta algorithm—a robust feature selection method based on Random Forest—was employed to identify the most predictive variables for LOHS from perioperative parameters. Four machine learning models (Linear Model, Random Forest, XGBoost, and K-Nearest Neighbors) were built using Boruta-confirmed features and evaluated via 10-fold cross-validation.

**Results:**

General anesthesia (GA) was associated with significantly longer LOHS (15.93 ± 4.04 days) compared to intrathecal anesthesia (IA) (14.31 ± 4.09 days, *p* = 0.012). The Boruta algorithm confirmed anesthesia method as an important predictor, along with age and preoperative hospitalization duration. While the Linear Model offered the best interpretability (mean *R*^2^ = 0.475), XGBoost showed a strong ability to model nonlinear patterns. Subgroup analyses revealed that GA was linked to prolonged anesthesia time, higher blood loss, and increased transfusion requirements, factors that are collectively associated with extended hospitalization.

**Conclusion:**

Despite the limited sample size, the use of Boruta feature selection ensured model focus on clinically meaningful predictors. We identify anesthesia method as a key modifiable factor associated with LOHS after TKA. These data-driven insights support the preferential consideration of IA for eligible patients in similar clinical contexts to potentially shorten hospital stays and optimize resource use, demonstrating the value of explainable machine learning in small-sample clinical studies.

## Introduction

Knee arthritis stands as one of the most prevalent chronic conditions in orthopedics, posing a substantial burden on global healthcare systems and impairing functional independence. With its global incidence on the rise (1), the optimization of management strategies has become increasingly urgent. Total knee arthroplasty (TKA) is a well-established intervention for knee arthritis, proven to effectively alleviate pain and restore joint function (2, 3). However, prolonged waiting times for TKA, exacerbated by limited hospital resources, can lead to significant treatment delays. In this context, optimizing the length of hospital stay (LOHS) has emerged as a critical strategy (4), as shortening LOHS not only enhances healthcare resource efficiency to accommodate more patients but also correlates with reduced postoperative complications and lower medical costs (5, 6). Notably, formal cost-utility analyses directly comparing intrathecal anesthesia (IA) and general anesthesia (GA) remain scarce while the economic benefits of shorter LOHS are quantifiable. Australian epidemiological data demonstrate a marginal cost of AUD 216 per general ward day reflecting the real opportunity cost of bed occupancy (7), and U.S. Medicare data link prolonged hospitalization due to complications to approximately USD 16,000 in excess expenditure per case (8). Shorter stays further drive episode-of-care savings by minimizing inpatient costs and the need for costly post-acute care.

Despite its clinical and economic importance, predicting LOHS remains challenging due to its multifactorial nature, involving patient demographics, comorbidities, surgical techniques, and perioperative management. Among these, the choice of anesthesia technique is particularly relevant. GA can lead to postoperative complications such as nausea, vomiting, respiratory depression from incomplete drug metabolism, and postoperative cognitive dysfunction. In contrast, IA carries risks of complications from difficult or failed puncture, as well as post-dural puncture headache, epidural hematoma, and infection (9). Such complications may contribute to prolonged hospitalization. Nevertheless, the specific impact of anesthesia type on LOHS remains controversial. While Harsten et al. reported that IA was associated with a shorter LOHS and reduced nausea, vomiting, and dizziness compared to GA (10), Mark et al. found no significant difference in LOHS between the two techniques (11). Even meta-analyses struggle to deliver consistent insights, as regional anesthesia, is statistically associated with modestly shorter LOHS for TKA and other major orthopedic surgeries, while the evidence is characterized by high heterogeneity. These discrepancies stem from inadequate confounding control in individual studies and methodological variations across research, including differing surgical populations and inconsistent definitions of anesthesia or LOHS (12–15). This highlights the need for a rigorous, data-driven approach to clarify anesthesia’s specific role in LOHS outcomes.

Traditional statistical methods often fall short in systematically evaluating variable importance and capturing potential subtle associations among predictors as they often require manual variable selection that may introduce subjective bias. In contrast, machine learning (ML) algorithms paired with the robust Boruta feature selection algorithm not only quantify the relative importance of each predictor but also eliminate redundant variables to avoid overfitting. This targeted and objective feature selection is critical for our core aim of disentangling anesthesia’s impact on LOHS amid multiple correlated perioperative factors.

Therefore, this study aims to develop ML models for predicting LOHS following TKA, utilizing Boruta for feature selection to improve predictive accuracy. Furthermore, we seek to elucidate the impact of anesthesia method on LOHS, addressing existing controversies through robust feature selection and multivariate adjustment. By integrating predictive modeling with causal inference, this research aims to offer practical insights for optimizing perioperative care in TKA.

## Materials and methods

### Study design and patient enrollment

**FIGURE 1:**
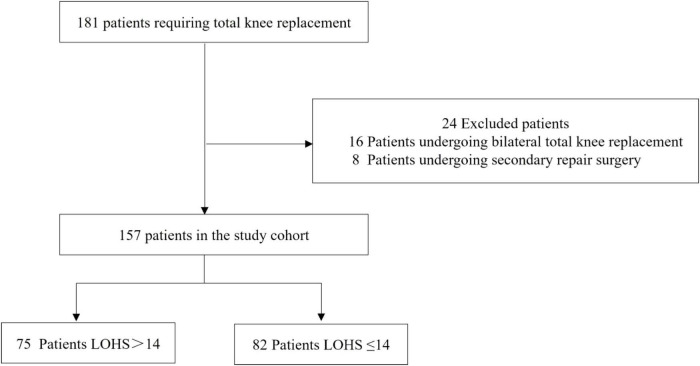
Flow chart. LOHS, length of hospital stay.

This retrospective study reviewed data from 181 patients who underwent TKA at Tangdu Hospital, Air Force Military Medical University, between January 1, 2020, and October 1, 2021. Patients who underwent bilateral TKA (*n* = 16) or secondary revision TKA (*n* = 8) were excluded. Consequently, a total of 157 patients receiving primary unilateral TKA were ultimately included (Figure 1). All data were independently collected by two anesthesiologists following a unified manual to ensure inter-rater reliability, with discrepant records resolved by a senior anesthesiologist. Missing data (<5%) were handled via multiple imputation, and pooled results were used for final analysis to minimize bias.

### Variables and observation indicators

The primary outcome was the LOHS, defined as the duration from admission to discharge. A prolonged LOHS was defined as > 14 days, based on both the average LOHS at our institution and the median LOHS in our sample.

Data collection encompassed demographic characteristics (age, gender, body mass index), preoperative comorbidities (cardiovascular, respiratory, endocrine, neurological, and other systemic diseases), and intraoperative parameters. The latter included preoperative hemoglobin, American Society of Anesthesiologists (ASA) classification, anesthesia method (GA or IA), operation time, anesthesia time, intraoperative urine output, blood loss, and volumes of crystalloids, colloids, and blood products administered.

### Anesthesia protocols

Anesthesia type for primary unilateral TKA was determined during the preoperative anesthesia evaluation at our center, based on patient-specific factors including preoperative comorbidities, ASA physical status classification, patient preference, and contraindications to neuraxial anesthesia, and was not influenced by anticipated surgical complexity for routine primary TKA procedures. For patients under GA, standardized intravenous-inhalation combined anesthesia was administered. Induction was performed using propofol (2–3 mg/kg) or etomidate (0.3 mg/kg), sufentanil (5 μg/kg), midazolam (2 mg), and rocuronium (0.9–1 mg/kg). Anesthesia was maintained with remifentanil (0.10–0.30 μg/kg/min), propofol (3–5 mg/kg/h), and sevoflurane (1–2%), with dosages titrated according to intraoperative vital signs.

For IA, combined spinal-epidural anesthesia was employed. A subarachnoid block was initiated with 10–15 mg of ropivacaine, followed by epidural administration of an additional 50 mg ropivacaine for procedures expected to exceed 4 h. Intraoperative sedation was provided using midazolam (2 mg IV) or dexmedetomidine (0.5 μg/kg/h). For postoperative analgesia, a periarticular cocktail injection (ropivacaine 150 mg + dexamethasone 5 mg) was administered. All patients received patient-controlled intravenous analgesia with standardized drug formulations, with doses adjusted by body weight.

### Ethics

For controversial data, another anesthesiologist re-screened the data based on the original data. This study was approved by the Ethics Committee of Tangdu Hospital of Air Force Military Medical University with the protocol number K202308/16. The personal information of patients was hidden. Because the study was retrospective and did not involve an intervention with patients, the Ethics Committee considered granting an informed consent waiver and therefore informed consent was not required. This study has been registered in the Chinese clinical trial registry (ChiCTR2500096147).

### Statistical analysis

Continuous variables are presented as mean ± standard deviation (SD), and categorical variables as percentages. The normality of data distribution was assessed using the Shapiro-Wilk test. Group comparisons (based on LOHS) were performed using the independent *t*-test (for normally distributed data), the Mann-Whitney U test (for non-normality data), or the χ^2^ test (for categorical variables).

Feature selection for predicting LOHS was performed using the Boruta algorithm, which iteratively compares the importance of original features against randomly permuted shadow features over 100 runs. Features were classified as confirmed, tentative, or rejected, with only confirmed important variables retained for predictive modeling. To further verify the stability of feature selection, we additionally conducted Boruta iterations with 1,000 runs, core predictive variables were consistently confirmed across both 100 and 1,000 runs parameter combinations, demonstrating reliable feature selection stability.

Predictive models for LOHS were developed using four machine learning algorithms: Linear Regression, Random Forest, Extreme Gradient Boosting (XGBoost), and K-Nearest Neighbors (KNN). All models were evaluated via 10-fold cross-validation. Performance was assessed using the root mean squared error (RMSE), coefficient of determination (R^2^), and mean absolute error (MAE). Model stability was visualized using boxplots of cross-validation results, and prediction accuracy was examined via scatter plots of predicted versus true values.

Based on Boruta results implicating anesthesia method, subgroup analyses were conducted. Significant continuous variables from univariate analyses were transformed into categorical variables to explore interaction effects.

All statistical analyses and machine learning modeling were conducted in R software (version 4.2.2). Key packages included Boruta for feature selection, caret for model training, and xgboost for implementing the XGBoost algorithm. A two-sided *p* < 0.05 was considered statistically significant.

## Results

### Characteristics of patients

A total of 157 patients were included in this study. The cohort was stratified by LOHS, with 82 patients (52.2%) in the shorter LOHS group (mean LOHS: 11.58 ± 2.13 days) and 75 patients (47.8%) in the longer LOHS group (mean LOHS: 19.54 ± 3.70 days). The shorter LOHS group (≤14 days) comprised 28 males and 54 females with a mean age of 67.40 years, while the longer LOHS group ( > 14 days) included 15 males and 60 females with a mean age of 66.39 years.

The distribution of anesthesia methods differed significantly between the two groups. GA was administered to 37 patients (49.3%) in the longer LOHS group, compared to 21 patients (25.6%) in the shorter LOHS group. Conversely, the proportion of patients receiving IA was significantly higher in the shorter LOHS group (74.7% vs. 50.7%, *p* = 0.002). No significant differences were observed between the groups in terms of preoperative comorbidities (including cardiovascular, respiratory, endocrine, nervous system, and other diseases) or other perioperative parameters such as ASA classification, preoperative hemoglobin levels, anesthesia duration, operation time, intraoperative blood loss, urine output, or volumes of crystalloid and colloid administered (Table 1).

**TABLE 1:** Characteristics of patients under TKA.

Characteristic	Shorter LOHS (*n* = 82)	Longer LOHS (*n* = 75)	*p*
Age	67.40 ± 7.78	66.39 ± 7.44	0.313
Gender (male)	28.00 (34.1%)	15.00 (20.1%)	0.071
BMI	25.27 ± 3.04	25.55 ± 3.77	0.976
Length of hospital stay, day	11.58 ± 2.13	19.54 ± 3.70	< 0.001*
Day of operative			0.130
Monday	6.00 (7.3%)	14.00 (18.7%)	
Tuesday	18.00 (22.0%)	16.00 (21.3%)	
Wednesday	14.00 (17.1%)	14.00 (18.7%)	
Thursday	22.00 (26.8%)	14.00 (18.7%)	
Friday	21.00 (24.4%)	15.00 (20.0%)	
Saturday	1.00 (1.2%)	2.00 (2.7%)	
Sunday	0.00 (0%)	0.00 (0%)	
**Preoperative comorbidities**			
Cardiovascular diseases	39.00 (47.6%)	35.00 (46.7%)	0.911
Respiratory diseases	4.00 (4.9%)	1.00 (1.3%)	0.369
Endocrine system diseases	9.00 (11.0%)	10.00 (13.3%)	0.417
Nervous system diseases	10.00 (12.2%)	11.00 (14.7%)	0.815
Other diseases	33.00 (40.2%)	27.00 (36.0%)	0.624
Intraoperative			0.126
ASA classification (I)	5.00 (6.1%)	0.00 (0.0%)	
ASA classification (II)	69.00 (84.1%)	68.00 (90.7%)	
ASA classification (III)	8.00 (9.8%)	7.00 (9.3%)	
Anesthesia method			0.002*
GA	21.00 (25.6%)	37.00 (49.3%)	
IA	61.00 (74.4%)	38.00 (50.7%)	
Preoperative hemoglobin, g/L	128.45 ± 14.43	127.26 ± 15.99	0.581
Anesthesia time, min	177.36 ± 55.50	185.76 ± 77.05	0.910
Operation time, min	130.66 ± 52.95	141.59 ± 73.05	0.622
Intraoperative blood loss, mL	256.47 ± 222.91	324.41 ± 290.90	0.146
Intraoperative urine output, mL	521.76 ± 296.25	534.95 ± 299.07	0.697
Intraoperative crystal input, mL	1054.12 ± 327.53	1055.91 ± 377.77	0.984
Intraoperative colloid input, mL	382.35 ± 251.75	426.63 ± 273.71	0.318
Intraoperative blood input, ml	131.76 ± 280.40	202.17 ± 358.87	0.177

Values are presented as mean ± SD. ASA, American society of anesthesiologists; BMI, Body mass index; LOHS, Length of hospital stay; GA, General anesthesia; IA, intravertebral anesthesia; TKA, total knee arthroplasty.

*p < 0.05.

### Risk factors for LOHS after TKA surgery

**FIGURE 2:**
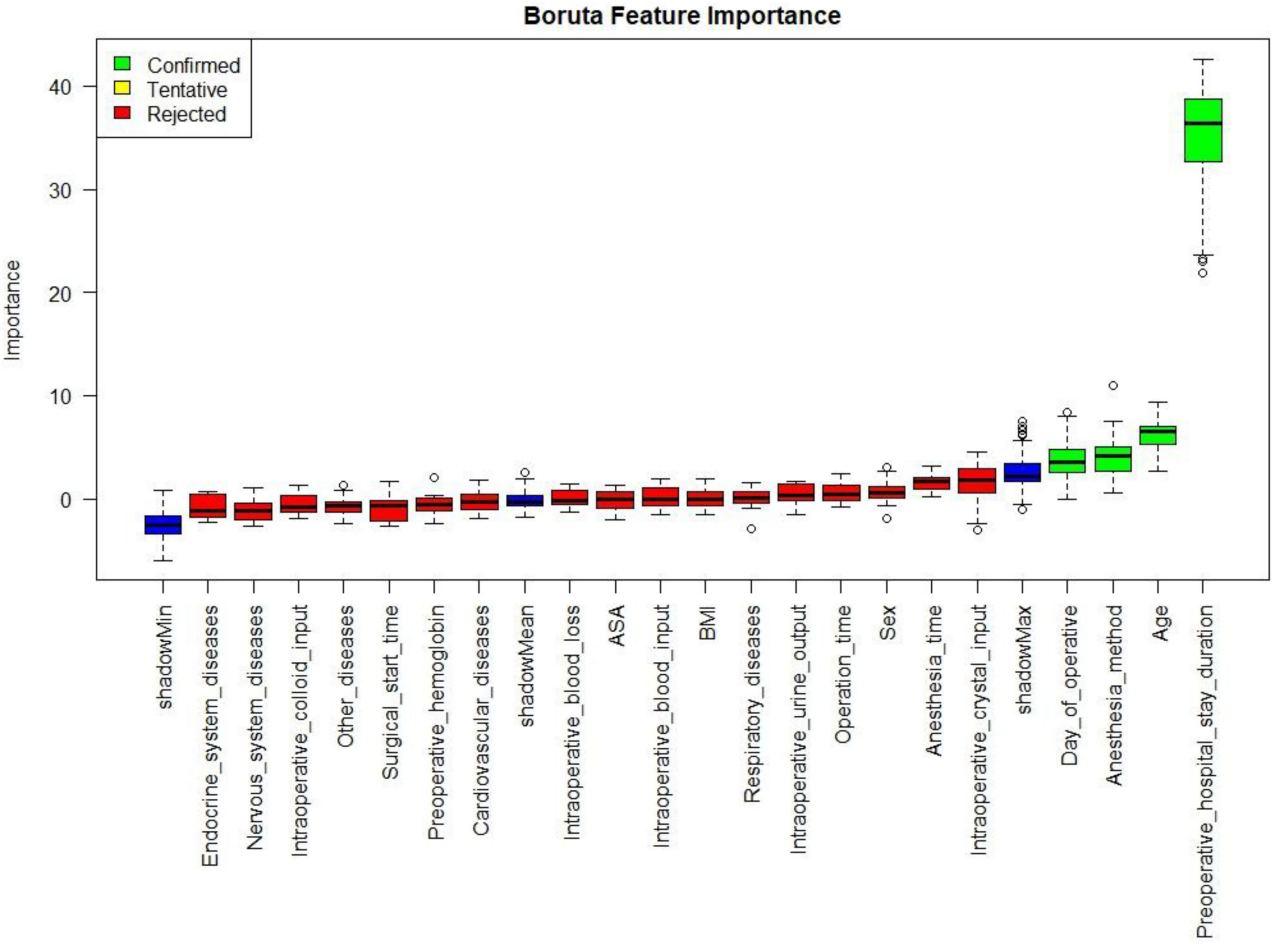
Boruta algorithm for feature importance in hospital length of stay prediction. The Boruta algorithm was used to assess the predictive importance of potential risk factors for LOHS in patients with TKA. The horizontal axis denotes variable names, and the vertical axis shows the random forest-derived Z-score importance metric. Variables are categorized by color: green boxes indicate confirmed important features, age, preoperative hospital stay duration, day of operative, and anesthesia method. Yellow boxes represent tentative features, while red boxes denote rejected non-informative features. LOHS, Length of hospital stay; TKA, Total knee arthroplasty.

The Boruta algorithm was employed to systematically evaluate the predictive importance of various candidate features for LOHS. This method iteratively compares the importance of actual features against randomly generated shadow features using a random forest-derived Z-score metric. With the application of Bonferroni correction (mcAdj = TRUE, *p* = 0.01) to control for multiple testing, four variables were confirmed as important predictors: age, preoperative hospital stay duration, day of surgery, and anesthesia method (Figure 2).

### Performance of machine learning models for predicting LOHS using Boruta-selected features

**FIGURE 3:**
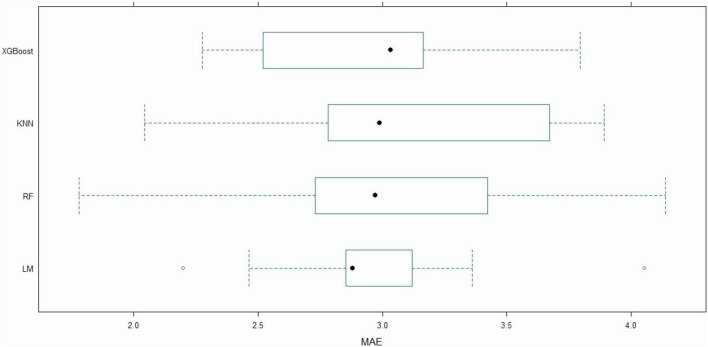
Boxplot of MAE among machine learning models. Distribution of MAE for LM, XGBoost, RF, and KNN models during 10-fold cross-validation. The box denotes the IQR, and open circles represent outliers. IQR, Interquartile range; KNN, K-Nearest neighbors; LM, Linear regression; MAE, Mean absolute error; RF, Random forest; XGBoost, Extreme gradient boosting.

**FIGURE 4:**
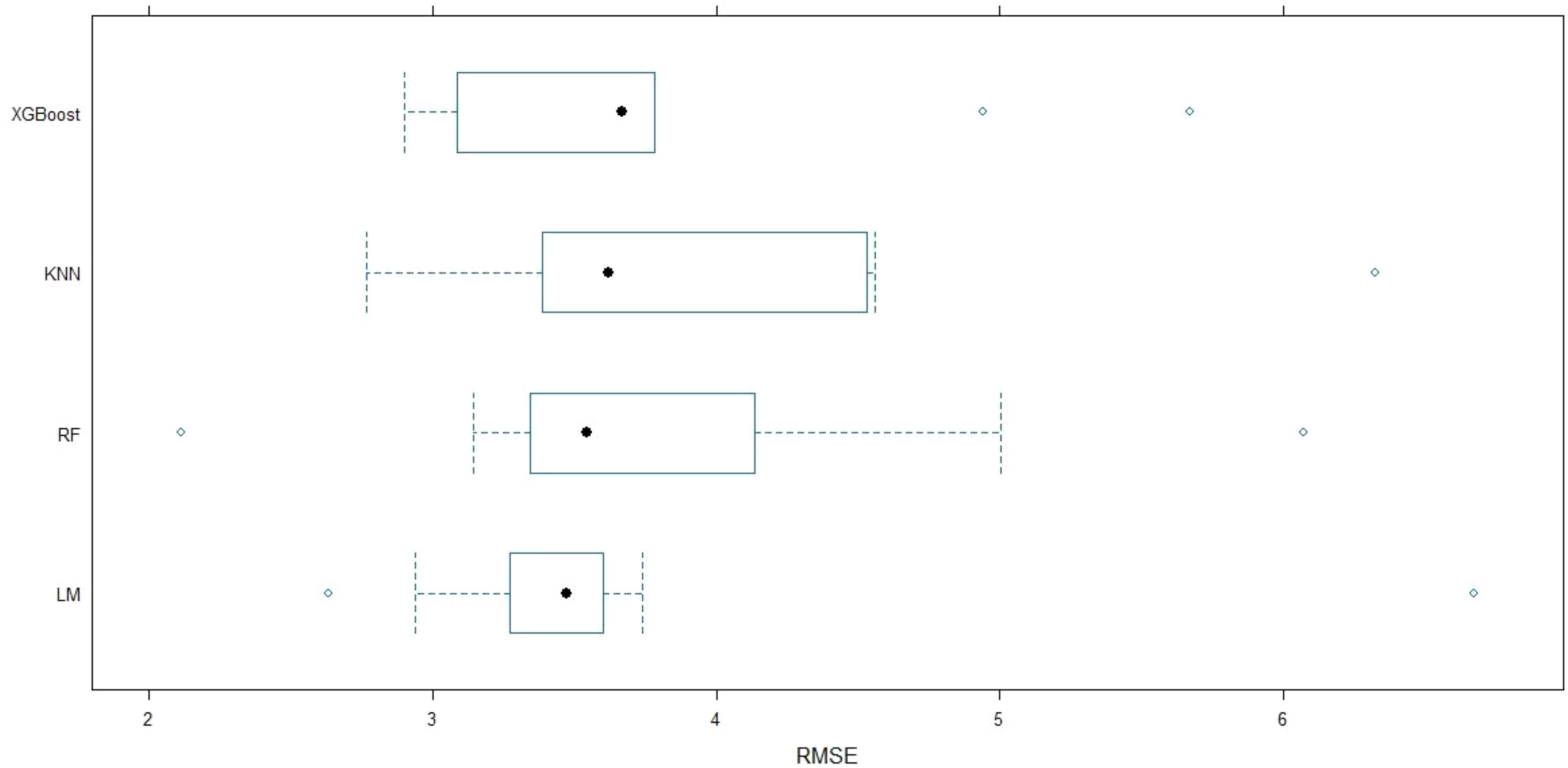
Boxplot of RMSE among machine learning models. Distribution of RMSE for LM, XGBoost, RF, and KNN models over 10-fold cross-validation. The box illustrates the IQR, and open circles denote outliers. IQR, Interquartile range; KNN, K-Nearest neighbors; LM, Linear regression; RF, Random forest; RMSE, Root mean squared error; XGBoost, Extreme gradient boosting.

**FIGURE 5:**
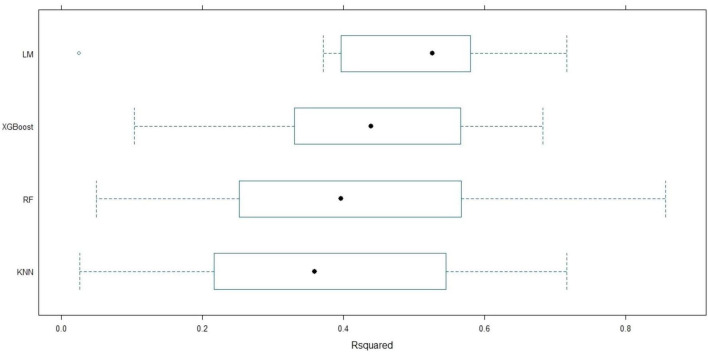
Boxplot of R-squared among machine learning models. Distribution of the R^2^ for LM, XGBoost, RF, and KNN models across 10-fold cross-validation. The box represents the IQR, and open circles indicate outliers. IQR, Interquartile range; KNN, K-Nearest neighbors; LM, Linear regression; RF, Random forest; XGBoost, Extreme gradient boosting.

After feature selection with the Boruta algorithm, four machine learning models—LM, RF, KN), and XGBoost—were trained using the confirmed features to predict LOHS. Given the limited sample size, a 10-fold cross-validation strategy was adopted to mitigate overfitting and evaluate model generalizability, with performance assessed via RMSE, MAE, and R^2^ (Figures 3–5). To further assess the variability and stability of model performance, we conducted 1,000 bootstrap resamples. The 95% confidence intervals (CIs) for RMSE, MAE, and R^2^ were calculated based on these 1,000 bootstrap iterations, enabling robust evaluation of model performance consistency.

Among the models, the LM demonstrated the strongest explanatory power, achieving a mean R^2^ of 0.475 (95% CI: 0.36–0.58). The XGBoost model exhibited the lowest prediction errors, with mean RMSE and MAE values of 3.789 (95% CI: 3.33–4.24) and 2.955 (95% CI: 2.69–3.21), respectively, indicating its superior capability in capturing nonlinear relationships between predictors and LOHS. The RF model showed intermediate performance (mean R^2^: 0.410, 95% CI: 0.29–0.56; RMSE: 3.837, 95% CI: 3.13–4.54; MAE: 3.075, 95% CI: 2.66–3.40), while the KNN model performed the poorest, with the highest mean RMSE (3.943, 95% CI: 3.42–4.65) and the lowest R^2^ (0.372, 95% CI: 0.25–0.46).

**FIGURE 6:**
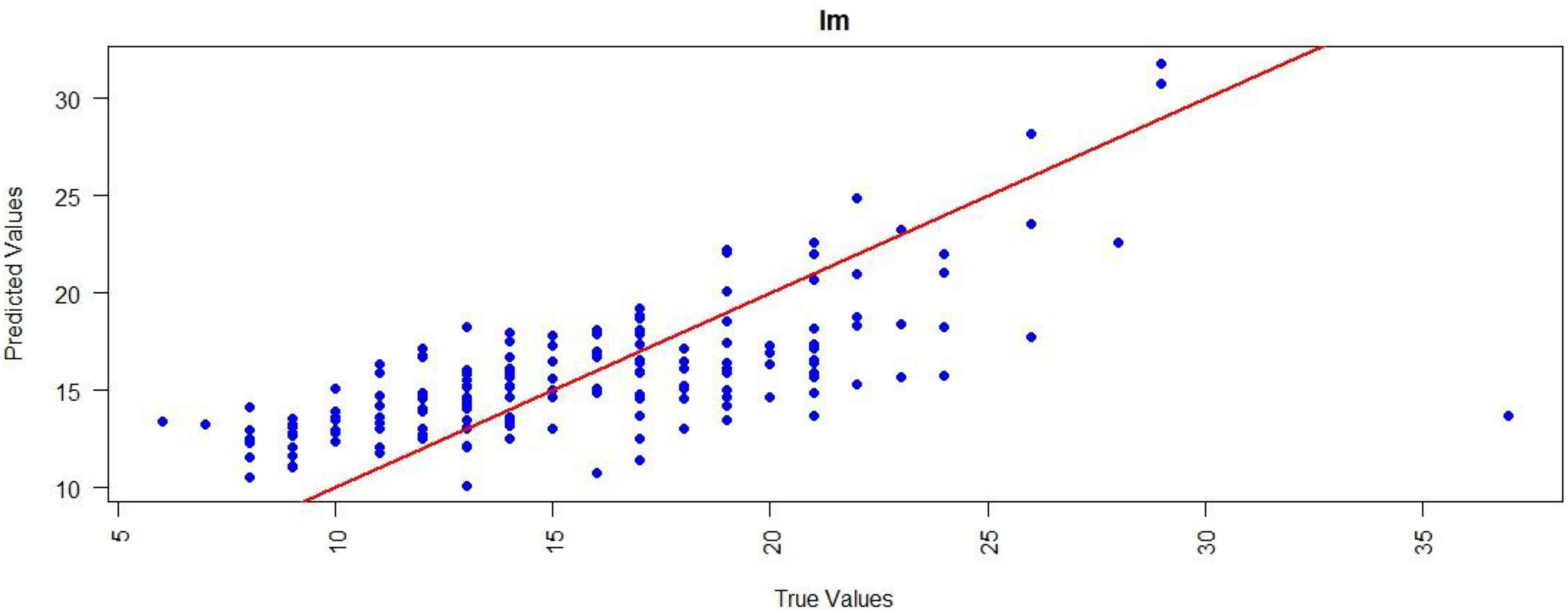
Performance evaluation of LM for LOHS prediction. Scatter plot illustrating the relationship between predicted and true LOHS. Points deviate more from the y = x diagonal at higher LOHS. LM, Linear regression; LOHS, Length of hospital stay.

**FIGURE 7:**
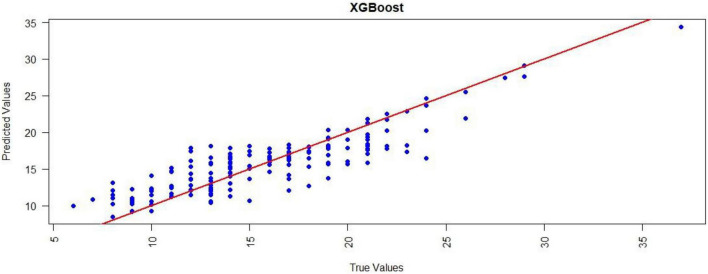
Performance evaluation of XGBoost for LOHS prediction. Scatter plot illustrating the relationship between predicted and true LOHS. Points deviate more from the y = x diagonal at higher LOHS. LOHS, Length of hospital stay; XGBoost, Extreme gradient boosting.

Scatter plots of predicted versus true LOHS values were generated to visually complement the quantitative metrics for the LM and XGBoost models (Figures 6, 7). For the LM, data points generally followed the y = x diagonal but displayed increased dispersion at higher LOHS values (>25 days), suggesting reduced prediction accuracy for extended stays. In contrast, the XGBoost model showed tighter clustering of points along the diagonal across the entire LOHS range, maintaining better prediction accuracy even at extreme values (>30 days), which underscores its robustness in modeling complex nonlinear patterns.

### Subgroup analysis of the effect of different anesthesia type on LOHS

Comparisons of general conditions and perioperative variables between the IA and GA groups are summarized in Table 2. The GA group demonstrated significantly longer anesthesia time (209.52 ± 68.06 vs. 165.78 ± 48.33 min, *p* < 0.001), longer operation time (161.84 ± 64.79 vs. 120.87 ± 45.70 min, *p* < 0.001), and greater intraoperative blood loss (395.69 ± 313.62 vs. 220.91 ± 179.19 mL, *p* = 0.001). Additionally, the GA group exhibited higher intraoperative urine output (611.21 ± 331.01 vs. 486.36 ± 288.10 mL, *p* = 0.010), greater crystalloid infusion (1210.34 ± 336.49 vs. 950.51 ± 297.40 mL, *p* < 0.010), and increased blood transfusion volume (273.68 ± 377.27 vs. 88.89 ± 212.32 mL, *p* < 0.001).

**TABLE 2:** The difference in intraoperative variables between IA and GA patients.

Characteristic	IA (*n* = 99)	GA (*n* = 58)	*p*
Age	66.95 ± 7.40	67.60 ± 6.57	0.348
Gender (male)	30.00 (30.30%)	13.00 (22.41%)	0.285
BMI	25.18 ± 3.09	25.81 ± 3.20	0.296
Length of hospital stay, day	14.31 ± 4.09	15.93 ± 4.04	0.012*
Length of hospital stay<14	61.00 (61.6%)	21.00 (36.2%)	0.002*
ASA classification			0.671
I	3.00 (3.0%)	2.00 (3. 5%)	
II	88.00 (88. 9%)	49.00 (84.5%)	
III	8.00 (8.1%)	7.00 (12.1%)	
Preoperative hemoglobin, g/L	128.25 ± 13.72	127.10 ± 14.67	0.696
Anesthesia time, min	165.78 ± 48.33	209.52 ± 68.06	< 0.001*
Operation time, min	120.87 ± 45.70	161.84 ± 64.79	< 0.001*
Intraoperative blood loss, mL	220.91 ± 179.19	395.69 ± 313.62	0.001*
Intraoperative urine output, ml	486.36 ± 288.10	611.21 ± 331.01	0.010*
Intraoperative crystal input, ml	950.51 ± 297.40	1210.34 ± 336.49	<0.001*
Intraoperative colloid input, ml	391.41 ± 260.40	447.37 ± 244.30	0.172
Intraoperative blood input, mL	88.89 ± 212.32	273.68 ± 377.27	< 0.001*

Values are presented as mean ± SD. ASA, American society of anesthesiologists; BMI, Body mass index; GA, General anesthesia; IA, intravertebral anesthesia.

*p < 0.05.

Several continuous variables—including operation time (cut-off: ≥ 110 min), anesthesia time ( ≥ 150 min), intraoperative blood loss ( ≥ 200 mL), urine output (≥500 mL), crystalloid input ( ≥ 1,000 mL), and blood transfusion volume ( > 0 mL)—were dichotomized based on median values. Compared to IA, GA was associated with longer LOHS in the presence of prolonged anesthesia time (OR 3.19, 95% CI: 1.36–7.49, interaction *p* = 0.014), higher intraoperative blood loss (OR 4.40, 95% CI: 1.39–14.26, interaction *p* = 0.010), and greater intraoperative blood transfusion (OR 2.61, 95% CI: 0.68–10.06, interaction *p* = 0.028). After Benjamini-Hochberg (BH) correction for multiple comparisons (FDR = 0.05), GA was significantly associated with longer LOHS in patients with prolonged anesthesia time (adjusted *p* = 0.042) and higher intraoperative blood loss (adjusted *p* = 0.042). A marginal trend was observed for greater intraoperative blood transfusion (adjusted *p* = 0.056) (Table 3).

**TABLE 3:** The relationship of method of anesthesia and LOHS, stratified by various anesthesia characteristics.

Characteristic	IA (reference)	GA (OR, 95% CI)	P for interaction	Adjusted P
Anesthesia time ≥ 150 min			0.014*	
Yes	1 (reference)	3.19 (1.36–7.49)		0.042*
No	1 (reference)	2.57 (0.75–8.77)		
Operation time ≥ 110 min			0.108	
Yes	1 (reference)	2.76 (1.10–6.97)		
No	1 (reference)	4.03 (1.29–12.63)		
Intraoperative blood loss ≥ 200 mL			0.010*	
Yes	1 (reference)	4.40 (1.39–14.26)		0.042*
No	1 (reference)	2.07 (0.84–5.09)		
Intraoperative urine output ≥ 500 mL			0.092	
Yes	1 (reference)	2.83 (0.98–8.12)		
No	1 (reference)	2.96 (1.19–7.31)		
Intraoperative crystal input ≥ 1,000 mL			0.812	
Yes	1 (reference)	1.20 (0.29–5.02)		
No	1 (reference)	4.63 (1.93–11.11)		
Intraoperative blood input			0.028*	
Yes	1 (reference)	2.61 (0.68–10.06)		0.058
No	1 (reference)	2.57 (1.14–5.76)		

Values are presented as mean ± SD. GA, General anesthesia; LOHS, Length of hospital stay; OR, Odd ratios; IA, intravertebral anesthesia.

*p < 0.05.

## Discussion

In this study, we identified a significant association between anesthesia method and LOHS following TKA, with GA linked to prolonged LOHS compared to IA. Validation through the Boruta algorithm confirmed anesthesia method as a key predictor of LOHS, alongside patient demographics and perioperative variables. Notably, the Boruta algorithm identifies variables with predictive importance for the outcome. The confirmed key features in this study reflect their predictive contribution to LOHS, and no causal inferences can be drawn from these results given the retrospective observational design of our study. Among the machine learning models evaluated, the LM demonstrated the strongest explanatory power and the lowest prediction error, enabling direct interpretation of anesthesia’s effect on LOHS. Although XGBoost exhibited a slightly lower R^2^, it effectively captured nonlinear relationships in the data. Notably, GA was associated with longer anesthesia times, greater intraoperative blood loss, and higher transfusion volumes—factors that collectively help explain the extended LOHS observed in these patients. While the linear model performed well, our ML framework adds value via Boruta’s unbiased feature selection, XGBoost’s capture of nonlinear anesthesia-LOHS relationships, and multi-model validation of core findings. The moderate R^2^ reflects LOHS’s multifactorial nature, with our focus on identifying modifiable predictors (e.g., anesthesia method).

Multiple studies have reported various factors associated with LOHS after TKA (16–20), including patient-related variables such as age, gender, and comorbidities, as well as surgery-related factors such as operative time, intraoperative fluid administration, and surgical approach. However, anesthesia-related factors remain underexplored, and existing evidence is often conflicting. This study strengthens the evidence for a strong association between anesthesia type and LOHS. Notably, the Boruta algorithm identified anesthesia method as one of the most important predictors.

Previous research has reported that regional anesthesia for postoperative pain management was associated with reduced LOHS in patients undergoing ankle open reduction and internal fixation (21). Similarly, Wang et al. found that patients receiving GA during hip and knee arthroplasty had longer hospital stays compared to those receiving regional anesthesia (11, 22). Patients undergoing GA during joint arthroplasty have also been associated with higher 30-day mortality and extended LOHS (23). Consistent with these findings, our study suggests that GA may be linked to longer LOHS compared to IA. Several potential pathways from external literature may explain this association. On one hand, GA is reported to have a higher incidence of short-term complications compared to IA. For instance, patients under GA often experience more severe postoperative pain in the later stages, requiring higher opioid doses, and exhibit significantly increased rates of nausea and vomiting (24, 25). Additionally, prior research has reported that GA may be associated with elevated levels of blood cortisol, insulin, and glucose, along with impaired cognitive function, all of which may contribute to prolonged hospitalization (26). Importantly, while our study did not directly measure these biochemical markers or cognitive outcomes, our subgroup analysis provides indirect supportive evidence. GA was significantly linked to prolonged anesthesia time, greater intraoperative blood loss, and increased transfusion requirements. These intraoperative factors may indirectly align with the proposed mechanisms from external studies, though they should not be construed as direct validation. In contrast, patients receiving IA experience fewer complications and side effects, which may facilitate earlier mobilization and functional recovery, thereby shortening LOHS (11, 27, 28). Collectively, these factors suggest that GA is associated with a range of postoperative sequelae that extend hospital stay.

Our interaction analysis further revealed that prolonged anesthesia duration, increased intraoperative blood loss, and higher transfusion requirements in GA patients were significantly associated with extended LOHS. Preoperative fasting combined with substantial intraoperative bleeding in GA patients can lead to hypovolemia, which may subsequently induce multi-organ dysfunction and increase postoperative morbidity, ultimately prolonging hospitalization (29, 30). Consequently, patients with low preoperative hemoglobin levels and substantial intraoperative blood loss often require timely transfusion, further complicating their recovery course.

Reducing LOHS not only lowers the risk of hospital-acquired complications such as deep vein thrombosis and nosocomial infections but also alleviates financial burdens and facilitates earlier reintegration into social and functional activities (31). In this study, machine learning–based feature selection with the Boruta algorithm confirmed anesthesia method as an important predictor of LOHS. While the linear regression model provided a direct quantification of the association between anesthesia type and LOHS, XGBoost uncovered subtler nonlinear relationships. For anesthesiologists and surgeons, such data-driven insights can support optimized resource allocation by identifying patients who would benefit most from targeted perioperative interventions, thereby directing limited healthcare resources to those at highest risk of prolonged hospitalization. A key potential confounding factor to consider is the relationship between anesthesia choice and surgical complexity. While GA was associated with longer operative time, increased intraoperative blood loss, and higher transfusion requirements in our cohort, anesthesia selection was made preoperatively based on patient-specific clinical factors rather than anticipated surgical difficulty. Additionally, the GA and IA groups exhibited balanced baseline characteristics, indicating no preoperative stratification of surgical complexity that would drive anesthesia choice. The Boruta algorithm further identified anesthesia method as an independent key predictor of LOHS. Collectively, these findings minimize the likelihood that anesthesia choice merely reflects anticipated surgical complexity, and support a direct association between anesthesia type and LOHS in our cohort.

While our findings highlight the potential benefits of IA for reducing LOHS, practical clinical translation requires acknowledgment of implementation barriers. IA is not universally applicable to all TKA patients, and GA remains a necessary, safe alternative for those ineligible for IA. Optimizing GA-related outcomes may serve as an alternative strategy to shorten LOHS. Clinicians should integrate our insights with patient-specific factors and institutional capabilities for personalized decision-making.

This study has several inherent limitations that should be considered. The relatively small sample size restricts the statistical power of subgroup analyses and model generalizability, with potential risks of unstable Boruta feature selection and inflated effect sizes; it may also account for the non-significant gender distribution trend observed between LOHS subgroups, though this trend did not confound the core association between anesthesia method and LOHS. Besides, although Boruta feature selection yielded stable results across 100 and 1,000 iteration runs, this Random Forest-based method has inherent limitations in our small dataset. There remains a potential risk of inflated feature importance estimates, and the accuracy of Random Forest-derived importance measures may be compromised by limited data volume, even with multi-run iterations to enhance the stability of feature selection. And then, the retrospective design introduces the possibility of unmeasured confounding factors correlated with anesthesia choice, hindering causal inference. The single-center setting further limits the external validity across diverse healthcare systems and populations. Additionally, the sole focus on LOHS as the primary outcome overlooks the assessment of long-term clinical indicators, precluding a comprehensive evaluation of anesthesia-related effects. Finally, we did not explore implementation barriers to IA adoption, conduct formal cost analyses, or distinguish between subcategories of GA and IA techniques, which further limits the practical applicability of our conclusions.

## Conclusion

Our findings have direct clinical implications for TKA perioperative care in our single-center military hospital setting. By validating anesthesia method as a key modifiable factor associated with LOHS through robust machine learning, this study provides a data-driven rationale for preferentially considering IA in eligible patients. These findings may not generalize to diverse populations or healthcare settings, where factors like surgeon expertise could influence outcomes. Adopting this evidence-based strategy can facilitate shorter-stay, patient-centered pathways, enhancing surgical efficiency and patient outcomes.

## Data Availability

The raw data supporting the conclusions of this article will be made available by the authors, upon reasonable request.

## References

[1] Price AJ Alvand A Troelsen A Katz JN Hooper G Gray A et al. Knee replacement. *Lancet.* (2018) 392:1672–82. 10.1016/s0140-6736(18)32344-4 30496082

[2] Lewis GN Rice DA McNair PJ Kluger M. Predictors of persistent pain after total knee arthroplasty: a systematic review and meta-analysis. *Br J Anaesth.* (2015) 114:551–61. 10.1093/bja/aeu441 25542191

[3] Fortier LM Rockov ZA Chen AF Rajaee SS. Activity recommendations after total hip and total knee arthroplasty. *J Bone Joint Surg Am.* (2021) 103:446–55. 10.2106/jbjs.20.00983 33337819

[4] Specht K Kjaersgaard-Andersen P Pedersen B. Patient experience in fast-track hip and knee arthroplasty–a qualitative study. *J Clin Nurs.* (2016) 25:836–45. 10.1111/jocn.13121 26708610

[5] Grosso MJ Neuwirth AL Boddapati V Shah RP Cooper H Geller J. Decreasing length of hospital stay and postoperative complications after primary total hip arthroplasty: a decade analysis from 2006 to 2016. *J Arthroplasty.* (2019) 34:422–5. 10.1016/j.arth.2018.11.005 30503306

[6] Healy WL Iorio R Ko J Appleby D Lemos DW. Impact of cost reduction programs on short-term patient outcome and hospital cost of total knee arthroplasty. *J Bone Joint Surg Am Volume.* (2002) 84:348–53. 10.2106/00004623-200203000-00003 11886902

[7] Page K Barnett AG Graves N. What is a hospital bed day worth? A contingent valuation study of hospital chief executive officers. *BMC Health Serv Res.* (2017) 17:137. 10.1186/s12913-017-2079-5 28196489 PMC5310013

[8] Cram P Lu X Li Y. Bundled payments for elective primary total knee arthroplasty. *Geriatric Orthopaedic Surg Rehabil.* (2014) 6:3–10. 10.1177/2151458514559832 26246946 PMC4318806

[9] Makito K Mouri H Matsui H Michihata N Fushimi K Yasunaga H. Spinal epidural hematoma and abscess after neuraxial anesthesia: a historical cohort study using the Japanese diagnosis procedure combination database. *Can J Anaesthesia.* (2021) 68:42–52. 10.1007/s12630-020-01827-w 33037571

[10] Harsten A Kehlet H Toksvig-Larsen S. Recovery after total intravenous general anaesthesia or spinal anaesthesia for total knee arthroplasty: a randomized trial. *Br J Anaesth.* (2013) 111:391–9. 10.1093/bja/aet104 23578860

[11] Neuman MD Rosenbaum PR Ludwig JM Zubizarreta JR Silber J. Anesthesia technique, mortality, and length of stay after hip fracture surgery. *JAMA.* (2014) 311:2508–17. 10.1001/jama.2014.6499 25058085 PMC4344128

[12] Elsenosy AM Hassan E Yousef AS Al-Alawi M Elbagory W. Impact of regional versus general anaesthesia on outcomes following total knee replacement: a systematic review and meta-analysis. *Cureus.* (2025) 17:e95445. 10.7759/cureus.95445 41311745 PMC12648450

[13] Johnson RL Kopp SL Burkle CM Duncan C Jacob AK Erwin P et al. Neuraxial vs general anaesthesia for total hip and total knee arthroplasty: a systematic review of comparative-effectiveness research. *Br J Anaesth.* (2016) 116:163–76. 10.1093/bja/aev455 26787787

[14] Xing J Wang J Liu G Jia Y. Effects of enhanced recovery after surgery on robotic radical prostatectomy: a systematic review and meta-analysis. *Gland Surg.* (2021) 10:3264–71. 10.21037/gs-21-699 35070886 PMC8749100

[15] Rele S Shadbolt C Schilling C Taylor N Dowsey M Choong P. The impact of enhanced recovery after surgery on total joint arthroplasty: protocol for a systematic review and meta-analysis. *JMIR Res Protoc.* (2021) 10:e25581. 10.2196/25581 33709944 PMC7998324

[16] Song X Xia C Li Q Yao C Yao Y Chen D et al. Perioperative predictors of prolonged length of hospital stay following total knee arthroplasty: a retrospective study from a single center in China. *BMC Musculoskelet Disord.* (2020) 21:62. 10.1186/s12891-020-3042-x 32005208 PMC6995082

[17] Basques BA Bell JA Sershon RA Della Valle C. The influence of patient gender on morbidity following total hip or total knee arthroplasty. *J Arthroplasty.* (2018) 33:345–9. 10.1016/j.arth.2017.09.014 28993087

[18] Wang HT Fafard J Ahern S Vendittoli PA Hebert P. Frailty as a predictor of hospital length of stay after elective total joint replacements in elderly patients. *BMC Musculoskelet Disord.* (2018) 19:14. 10.1186/s12891-018-1935-8 29338705 PMC5771036

[19] Martino J Peterson B Thompson S Cook JL Aggarwal A. Day of week and surgery location effects on stay length and cost for total joint arthroplasty: academic versus orthopaedic-specific hospital. *J Knee Surg.* (2018) 31:815–21. 10.1055/s-0037-1615299 29270951

[20] Newman JM Szubski CR Barsoum WK Higuera CA Molloy RM Murray TG. Day of surgery affects length of stay and charges in primary total hip and knee arthroplasty. *J Arthroplasty.* (2017) 32:11–5. 10.1016/j.arth.2016.06.032 27471211

[21] Alexander B Said E Gabriel RA. A national registry analysis of the association of perioperative regional anesthesia with hospital length of stay following open reduction and internal fixation of the ankle. *J Clin Anesth.* (2020) 67:110008. 10.1016/j.jclinane.2020.110008 32829110

[22] Wang X Li H Yuan C Zhao H. Association between type of anesthesia and length of hospital stay in primary unilateral total knee arthroplasty patients: a single-center retrospective study. *J Orthopaedic Surg Res.* (2021) 16:671. 10.1186/s13018-021-02817-4 34781975 PMC8591843

[23] Perlas A Chan V Beattie S. Anesthesia technique and mortality after total hip or knee arthroplasty: a retrospective, propensity score-matched cohort study. *Anesthesiology.* (2016) 125:724–31. 10.1097/aln.0000000000001248 27466030

[24] Pu X Sun J. General anesthesia vs spinal anesthesia for patients undergoing total-hip arthroplasty: a meta-analysis. *Medicine.* (2019) 98:e14925. 10.1097/md.0000000000014925 31008923 PMC6494405

[25] Macfarlane AJ Prasad GA Chan VW Brull R. Does regional anesthesia improve outcome after total knee arthroplasty? *Clin Orthop Relat Res.* (2009) 467:2379–402. 10.1007/s11999-008-0666-9 19130163 PMC2866929

[26] Edipoglu IS Celik F. The associations between cognitive dysfunction, stress biomarkers, and administered anesthesia type in total knee arthroplasties: prospective, randomized trial. *Pain Phys.* (2019) 22:495–507. 31561651

[27] Awad IT Cheung JJ Al-Allaq Y Conroy PH McCartney CJ. Low-dose spinal bupivacaine for total knee arthroplasty facilitates recovery room discharge: a randomized controlled trial. *Can J Anaesthesia.* (2013) 60:259–65. 10.1007/s12630-012-9867-5 23229869

[28] Foss NB Kristensen MT Kristensen BB Jensen PS Kehlet H. Effect of postoperative epidural analgesia on rehabilitation and pain after hip fracture surgery: a randomized, double-blind, placebo-controlled trial. *Anesthesiology.* (2005) 102:1197–204. 10.1097/00000542-200506000-00020 15915033

[29] Gan TJ Soppitt A Maroof M el-Moalem H Robertson KM Moretti E et al. Goal-Directed Intraoperative Fluid Administration Reduces Length of Hospital Stay after Major Surgery. *Anesthesiology.* (2002) 97:820–6. 10.1097/00000542-200210000-00012 12357146

[30] Yogendran S Asokumar B Cheng DC Chung FA. Prospective randomized double-blinded study of the effect of intravenous fluid therapy on adverse outcomes on outpatient surgery. *Anesth Analg.* (1995) 80:682–6. 10.1097/00000539-199504000-00006 7893018

[31] Walker JB Nguyen PL Schmidt UH Gabriel RA. Postoperative outcomes associated with neuraxial vs general anesthesia following bilateral total knee arthroplasty. *J Arthroplasty.* (2017) 32:3632–6. 10.1016/j.arth.2017.06.028 28709756

